# Prioritisation of patients on waiting lists for hip and knee arthroplasties and cataract surgery: Instruments validation

**DOI:** 10.1186/1472-6963-8-76

**Published:** 2008-04-08

**Authors:** Alejandro Allepuz, Mireia Espallargues, Montse Moharra, Mercè Comas, Joan MV Pons, Research Group on Support Instruments – IRYSS Network

**Affiliations:** 1 Quality in Health Care Area, Catalan Agency for Health Technology Assessment and Research, Carrer de Roc Boronat, 81-95 (segona planta) 08005, Barcelona, Spain; 2 Evaluation and Clinical Epidemiology Department, Hospital del Mar-IMAS, Passeig Marítim 25-29 08003, Barcelona, Spain; 3 CIBER Epidemiología y Salud Pública (CIBERESP), Spain

## Abstract

**Background:**

Prioritisation instruments were developed for patients on waiting list for hip and knee arthroplasties (AI) and cataract surgery (CI). The aim of the study was to assess their convergent and discriminant validity and inter-observer reliability.

**Methods:**

Multicentre validation study which included orthopaedic surgeons and ophthalmologists from 10 hospitals. Participating doctors were asked to include all eligible patients placed in the waiting list for the procedures under study during the medical visit. Doctors assessed patients' priority through a visual analogue scale (VAS) and administered the prioritisation instrument. Information on socio-demographic data and health-related quality of life (HRQOL) (HUI3, EQ-5D, WOMAC and VF-14) was obtained through a telephone interview with patients. The correlation coefficients between the prioritisation instrument score and VAS and HRQOL were calculated. For the reliability study a self-administered questionnaire, which included hypothetic patients' scenarios, was sent via postal mail to the doctors. The priority of these scenarios was assessed through the prioritisation instrument. The intraclass correlation coefficient (ICC) between doctors was calculated.

**Results:**

Correlations with VAS were strong for the AI (0.64, CI95%: 0.59–0.68) and for the CI (0.65, CI95%: 0.62–0.69), and moderate between the WOMAC and the AI (0.39, CI95%: 0.33–0.45) and the VF-14 and the CI (0.38, IC95%: 0.33–0.43). The results of the discriminant analysis were in general as expected. Inter-observer reliability was 0.79 (CI95%: 0.64–0.94) for the AI, and 0.79 (CI95%: 0.63–0.95) for the CI.

**Conclusion:**

The results show acceptable validity and reliability of the prioritisation instruments in establishing priority for surgery.

## Background

Traditionally, the only explicit system to prioritise patients awaiting surgery has been the timing of the patient's inclusion in the waiting list, although various studies show how different factors may in practise influence the waiting period [1-3]. The lack of explicit prioritisation criteria that may cause patients with the same level of need to have very different waiting periods and the negative health effects of delay of surgery, further reinforce the necessity to develop instruments which enable the waiting list to be ordered in line with patients' needs [2,4-8].

Several countries, principally New Zealand, Canada and the United Kingdom, have developed prioritisation instruments as the strategy for managing waiting lists according to the needs of the patients and the benefit expected from surgery [9]. Nevertheless, most of these instruments have included doctors and other health professionals views, whereas patients' or other social groups' preferences have had little or no direct input [4,10-13]. In Spain, in the Basc Country and in Catalonia, prioritisation instruments for hip and knee arthroplasties and cataract surgery have been elaborated [14-17]. In Catalonia, the prioritisation instruments developed by the Catalan Agency for Health Technology Assessment and Research (CAHTA) elicited general population, patients and close relatives, allied-health professionals and consultants preferences to establish surgical priority [16,17].

The development of prioritisation instruments should be accompanied by an evaluation of their capacity to measure the priority of patients awaiting surgery and of their reliability. The objectives of this study were to evaluate the construct validity and inter-observer reliability of the prioritisation instruments developed by CAHTA.

## Methods

This was a multicentre validation study which included patients scheduled for hip and knee arthroplasties and cataract surgery between June 2001 and June 2002 and May 2004 and March 2006 in 10 hospitals of different Spanish Autonomous Communities: 2 in Andalusia, 2 in Aragon, 2 in the Canary Islands and 4 in Catalonia. Orthopaedic surgeons and Ophthalmologists from these centres were invited to participate by recruiting and assessing the priority for surgery of their patients and answering a specific questionnaire to analyse the inter-observer reliability of the prioritisation instruments.

### CAHTA's prioritisation instruments

Conjoint analysis was used to develop point-count scoring instruments for setting priority. This technique has been used in health care to involve patients and the community in planning and developing healthcare services and to investigate priority of patients on waiting lists and differences on judgements among different stakeholders [18,19]. In a first stage, 4 focus and nominal groups consisting of general population, patients and close relatives, allied-health professionals (general practitioners, nurses, social workers, optometrists, and physiotherapists) and consultants (orthopaedic surgeons, rheumatologists, rehabilitators, ophthalmologists, and general practitioners) identified and selected priority criteria, and their levels were established by the research group. All possible combinations of criteria levels were generated with each combination becoming a patient scenario. In a second stage, participants were asked to rank the scenarios from the highest to the lowest priority for surgery. The score for the levels of each criterion was calculated from this ranking through a rank-ordered logit model. The prioritisation instruments consist of 7 criteria for arthroplasties and 6 for cataract surgery with 2 to 4 levels for each of the criteria. The overall priority score is derived from the sum of the scores in each criterion and it ranges between 0 – the lowest priority- and 100 – the highest priority – (Table 1) [16,17].

**Table 1:** Prioritisation instruments' criteria, levels and scores

	Prioritisation instruments	
	
Criteria	Hip and knee arthroplasty	Cataract surgery
Severity of the disease:		-mild (0)
-Arthroplasty: clinical and radiological exploration	-moderate (0)	-moderate (20)
-Cataract surgery: visual incapacity^1^	-severe (18)	-severe (35)
		-extremely severe (45)
Pain	-mild (0)	-
	-moderate (17)	
	-severe (33)	
Probability of recovery	-moderate (0)	-moderate (0)
	-high (4)	-high (6)
		-very high (7)
Difficulty in doing ADL^2^	-has some difficulty (0)	- has some difficulty (0)
	-has great difficulty (10)	- has great difficulty (11)
	-Unable to do most ADL (20)	- Unable to do most ADL (15)
Limitation on ability to work	-no/does not work (0)	- no/does not work (0)
	-yes (10)	-yes (14)
Has someone to look after the patient	-yes (0)	-yes (0)
	-no (9)	-no (11)
Be a care-giver	-no (0)	-no (0)
	-yes (6)	-yes (8)

^1 ^Best eye (BE) > 0.4 and worst eye (WE) >= 0.2 severity of the disease (SD) mild, BE > 0.4 and WE < 0.2 SD moderate, BE 0.2–0.4 and WE >= 0.2 or <0.2 SD severe, BE < 0.2 and WE >= 0.2 or <0.2 SD extremely severe

^2 ^ADL: activities of daily living

### Convergent and discriminant validity study

During the medical visit, participant doctors invited patients that were placed in the waiting list for hip and knee arthroplasty and cataract surgery to join the study. Patients where the prosthesis was exchanged or those with urgent operations were excluded. Before their recruitment, the patients signed an informed consent.

The participant doctors were asked to assess patients' priority for surgery during the medical visit through a 10-centimetre visual analogue scale (VAS) according to the average patient they usually attend – where 0 was the lowest priority and 10 the highest- and to administer the prioritisation instrument. To prevent the influence that the prioritisation criteria might have on a doctor's opinion on surgical priority (VAS), the VAS was answered before the prioritisation criteria were assessed.

Patients' information for the study was also collected through a telephone interview carried out by a pre-trained interviewer within the following 4 months after the medical visit. It yielded information on their socio-demographic characteristics (date of birth, sex, occupational status and level of education), overall and ocular comorbidities, previous arthroplasty or cataract surgery, perception of the difficulty caused by the condition (none, a little, moderate or a great deal) and self-perceived health status and functional capacity through 2 generic health-related quality of life (HRQOL) questionnaires – Health Utilities Index Mark 3 (HUI3) [20] and the EQ-5D [21]- and 1 disease specific – the Western Ontario McMaster Osteoarthritic Index (WOMAC) [22] for arthroplasties and the Visual Function Index VF-14 (VF-14) [23] for cataract surgery. Patients who did not answer by themselves were excluded from the analysis -124 in arthroplasties and 403 in cataract surgery.

### Convergent and discriminant validity analysis

Convergent and discriminant validity form part of what is known as construct validity. In the same way as in New Zealand and Canada, we used correlation analysis to assess the prioritisation instruments construct validity [24-27]. This method aims to find whether the construct developed – surgical priority-, presents higher correlation with aspects or instruments already validated to which they should be related theoretically (convergent validity), than with those that measure aspects that are not similar to the construct being evaluated (discriminant validity) [28]. Construct validity was evaluated only through correlation analysis. We did not use factor analysis to analyse construct validity due to the fact that our theoretical framework for the development of the prioritisation instruments did not contemplate a previous hypothetical structure.

Doctor's opinion on surgical priority (VAS) and patient's perception of the difficulty caused by the condition and HRQOL were the only constructs which we considered that could be related to the priority construct measured by CAHTA's instruments. Although the prioritisation instruments are not intended to measure HRQOL, this construct reflects patients' necessity of surgery. On the other hand, generic HRQOL questionnaires include different dimensions that should not be correlated with the priority for surgery construct. It was expected that the dimensions of mobility and pain (for arthroplasties) and vision (for cataracts) would be moderately correlated with the overall priority score. Conversely, no correlation was expected between the overall priority score and the dimensions of cognition, dexterity, hearing, emotion, anxiety, and vision in arthroplasties and, in cataracts, personal care and pain (Table 2). Convergent and discriminant validity analysis was only performed for the overall priority score. Priority construct is defined by the combination of a number of factors, but it is its final result – the overall priority score – what should be a valid measure.

**Table 2:** Instruments and constructs included in the convergent and discriminant analysis

	Surgical priority instruments			
	
	Hip/Knee arthroplasty		Cataract surgery	
	
Instruments	Convergent	Discriminant	Convergent	Discriminant
Doctors' opinions (VAS)	Yes	No	Yes	No
Patients' perceptions on difficulty	Yes	No	Yes	No
WOMAC	Yes	No	No	No
VF-14	No	No	Yes	No
HUI3^1^	-Overall score -Pain, Ambulation	Vision, Emotion, Dexterity, Hearing, Cognition	-Overall score -Vision, Ambulation	Pain, Emotion, Anxiety/depression, Dexterity, Hearing, Cognition
EQ-5D	-Overall score -Pain, Daily activities, Mobility, Self-care	Anxiety/depression	-Overall score -Daily activities, Mobility	Pain, Anxiety/Depression, Self-care

VAS: visual analogue scale; WOMAC: Western Ontario McMaster Osteoarthritis Index; VF-14: Visual Function Index VF-14; HUI3: Health Utilities Index Mark 3.

^1 ^The speech dimension was not included in the analysis as none of the patients had difficulty with it.

The mean, the standard deviation (SD) and the range of values observed for the prioritisation instruments, the VAS, the HUI3, the EQ-5D, the WOMAC and the VF-14 were calculated. For each instrument the percentages of patients who scored the worst possible status (floor effect) and the best possible status (ceiling effect) were also calculated. These values give information on the suitability of the instrument to the studied population. A percentage lower than 15% of floor and ceiling effects was considered acceptable [29]. A higher percentage makes the instrument useless, as it would not be possible to discriminate between patients.

Spearman and correlation coefficients and their confidence interval of 95% (CI95%) were calculated to assess convergent and discriminant validity of the overall priority score. Correlation coefficients values between 0.1 and 0.3 were regarded as weak correlation, those between 0.3 and 0.5 as moderate, and those equal to or higher than 0.5 as strong. Values below 0.1 were regarded as having no correlation [30].

### Reliability study and analysis

Reliability is the degree to which the instrument is free from random error, which could be due to the precision of the scale based on the homogeneity of its items (internal consistency), or to its reproducibility which would be dependent on the instrument's stability over time (test-retest reliability) or to the degree of agreement among administrators (inter-observer reliability) [31]. The criteria that make up the CAHTA's prioritisation instruments were chosen on the basis of the preferences of the participants in the development study [16,17]. Because of that, the analysis of the reliability of the prioritisation instruments was focused on evaluating their inter-observer reliability since a correlation between the instruments' criteria was not a priori expected.

A set of hypothetical scenarios was drawn up by one of the investigators (ME), based on information obtained from checking clinical histories of patients who were going to be operated on arthroplasties or cataract surgery. Each of these scenarios described the characteristics of different patient priority profiles. In the case of hip and knee arthroplasties, 11 scenarios were created, and 10 for cataract surgery [see Additional file 1]. These scenarios were included in a self-administered questionnaire together with the prioritisation instrument that was sent to the participating doctors via postal mail. The scenarios were evaluated independently by each doctor on the basis of the prioritisation instruments. To analyse the inter-observer reliability, the intraclass correlation coefficient (ICC) and its CI95% between doctors' priority assessments were calculated. An ICC of 0.7 or higher was considered acceptable for group comparisons, while 0.9 or higher for individual comparisons [31].

## Results

The total number of patients included in the study was 944 for hip and knee arthroplasties and 1,674 for cataract surgery. Table 3 shows the main characteristics of the study population.

**Table 3:** Characteristics of the study population

	Hip/knee arthroplasties	Cataract surgery
Number of patients	944	1,674
Average age in years (SD)	69.3 (8.9)	72.9 (8.9)
Sex (women) (%)	72.8	58.1
Bilateral affectation (%)	18.8	56.7
Affected joints (%)		
Hip (left/right)	27.7/30.6	-
Knee (left/right)	66.8/68.4	-
Presence of ocular comorbidity (%)^1^	-	14.9
Previous arthroplasty or cataract surgery (%)	25.5	25.8
Operated joints (%)		
Hip	28.2	-
Knee	71.5	-
General comorbidities(%)		
Arthrosis or rheumatism	98.3	67.4
Asthma or chronic bronchitis	13.1	19.5
Cancer or other malign tumour	4.0	5.5
Diabetes	15.0	22.3
Embolism or paralysis	5.8	6.8
Hip or femur fracture	2.9	2.8
Hypertension	54.2	51.5
Heart problems	16.3	20.9
Depression or nervous problems	31.5	24.3
Deafness	26.1	36.1
Circulatory disorders	46.6	41.9
Prostate disorders^2^	33.5	31.7
Womb/ovary disorders^2^	16.6	14.7
Varicose veins	41.7	32.3
Occupational status (%)		
Retired	35.7	49.2
Housewife	50.0	41.9
Other^3^	14.3	8.9
Educational level (%)		
Unable to read and write	5.2	5.2
Primary school or below	82.3	84.8
Secondary school certificate, upper primary, or professional training	8.2	5.5
Higher secondary studies or university degree	4.3	4.4

^1 ^Ocular comorbidities: ambliopy, senile macular degeneration, diabetic retinopathy, glaucoma, previous retinal detachment.

^2 ^Prostate disorders divided by the number of men, and womb/ovary disorders divided by the number of women.

^3 ^Other: working, without occupation, unemployed, studying.

### Convergent and discriminant validity

The mean overall priority score was 49.4 (SD: 22.0) for the arthroplasty instrument (AI) and 35.3 (SD: 23.0) for the cataract instrument (CI). In both instruments, both the floor and the ceiling effects were low, with the highest being the ceiling effect of the CI at 4.6%. The properties of all the instruments used and the scores are shown in Table 4.

**Table 4:** Instruments' properties and the scores of the study population: priority and HRQOL

		Instrument properties			Observed scores				
	
Instruments	Procedure	Possible range	Floor effect (%)	Ceiling effect (%)	N	Mean (SD)	Minimum	Median	Maximum
Prioritisation									
Prioritisation instrument	Arthroplasty	100-0	0.9	0.4	785	49.4 (22.0)	0	49.0	100
	Cataract		0.0	4.6	1,547	35.3 (23.0)	0	35.0	99
Doctor's opinion on surgical priority (VAS)	Arthroplasty	10-0	0.2	0.7	881	5.9 (1.9)	0	5.9	10
	Cataract		0.3	3.3	1,378	4.5 (2.3)	0	4.5	10
HRQOL									
HUI3^1^	Arthroplasty	0–1	0.0	0.5	196	0.57 (0.20)	0.12	0.58	1
	Cataract		0.0	2.8	569	0.69 (0.22)	0.08	0.70	1
EQ-5D^1^	Arthroplasty	-0,08–1	0.6	0.6	347	0.37 (0.19)	-0.08	0.27	1
	Cataract		0.0	23.0	934	0.69 (0.24)	0.01	0.70	1
WOMAC	Arthroplasty	96-0	0.2	0.3	938	53.9 (17.6)	0	55.0	96
Pain^2^		20-0	0.5	1.4	939	10.3 (4.0)	0	10.0	20
Stiffness^2^		8-0	43.8	1.9	938	2.3 (2.4)	0	2.0	8
Function^2^		68-0	0.2	0.8	939	41.3 (13.0)	0	42.0	68
VF-14	Cataract	0–100	0.3	5.0	1,669	59.9 (25.5)	0	60.4	100

HRQOL: health-related quality of life; VAS: visual analogue scale; HUI3: Health Utilities Index Mark 3; VF-14: Visual Function Index VF-14; WOMAC: Western Ontario McMaster Osteoarthritis Index.

^1 ^The utility functions used have been adapted to the Spanish population. References 20 and 21.

^2 ^WOMAC questionnaire dimensions.

**Figure 1:**
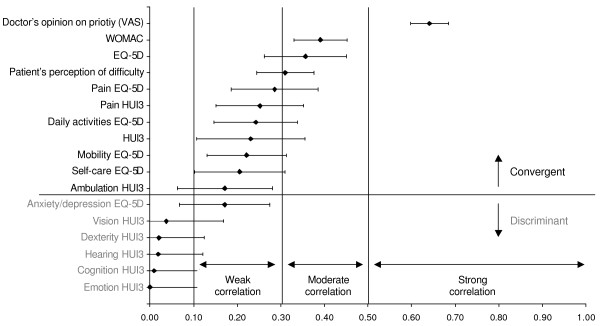
Overall priority score convergent and discriminant construct validity: hip and knee arthroplasties.

**Figure 2:**
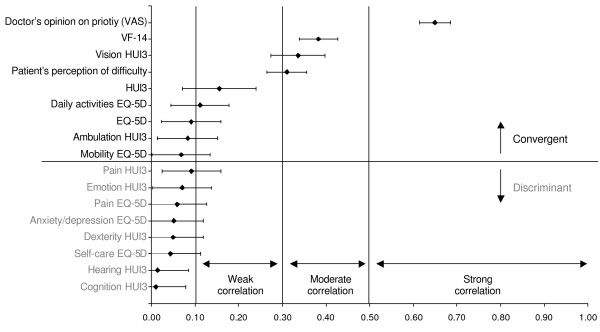
Overall priority score convergent and discriminant construct validity: cataract surgery.

For the construct validity, given that the direction of the correlations was in all cases as expected, absolute values of correlation coefficients were used, with the aim of facilitating their interpretation. The correlation of the overall priority score with the doctor's opinion on surgical priority (VAS) was strong both for AI (0.64, CI95%: 0.60–0.68) and for CI (0.65, CI95%: 0.61–0.69), whereas the correlation of the patient's perception of the difficulty caused by the condition was moderate both for AI (0.31, CI95%: 0.24–0.38) and for CI (0.31, CI95%: 0.26–0.36). Correlations were moderate between the WOMAC and the AI (0.39, CI95%: 0.33–0.45) and between the VF-14 and the CI (0.38, CI95%: 0.34–0.43). Correlation was low between the HUI3 and the AI (0.23, CI95%: 0.11–0.36) and the CI (0.16, CI95%: 0.07–0.24), and it was moderate between the EQ-5D and the AI (0.36, CI95%: 0.26–0.45). On the contrary to what was expected, the CI did not correlate with the EQ-5D, the ambulation dimension of the HUI3 and the mobility dimension of the EQ-5D (Figures 1 and 2).

Contrary to what was expected in the discriminant analysis, the AI presented a weak correlation with the anxiety/depression dimension of the EQ-5D. However, the other dimensions of the generic HRQOL questionnaires behaved as expected (Figures 1 and 2).

### Reliability

For the inter-observer reliability of the prioritisation instruments, 16 doctors participated in the AI analysis, while there were 49 CI participants. The number of evaluations for each scenario was 14 to 15 for the AI and 29 to 33 for the CI. The inter-observer ICC for the AI was 0.79 (CI 95%: 0.64–0.94), the same as for the CI (0.79, CI 95%, 0.63–0.95).

## Discussion

The prioritisation instruments for hip and knee arthroplasties and cataract surgery were designed to gather information on the necessity of surgery. Although there is no gold standard for comparing the priority construct, the doctor's opinion on surgical priority (VAS) and the patients' perception of the difficulty caused by the condition and HRQOL could be the nearest approximation. The strong correlation observed in the convergent analysis between the doctor's opinion on surgical priority (VAS) and the overall priority score indicates that the AI and CI are collecting the relevant aspects that doctors take into consideration when assessing the need for surgery. However, correlations with doctors' opinions were not higher because the criteria included in the prioritisation instruments and their scores not only reflect the opinion of medical specialists, but also of general population, patients and close relatives and allied-health professionals [16,17]. On the other hand, the prioritisation instruments include criteria related to patients' self-perception that could have also influenced the correlation between the overall priority score and doctors' opinion (VAS).

The disease specific HRQOL questionnaires, WOMAC and VF-14, and the patients' perception of the difficulty caused by their condition were also among the aspects that most strongly correlated with the overall priority score. This suggests that the prioritisation instruments are also able to collect patients' perceptions about how aspects of their daily life have been affected by their condition. Nevertheless, generic and specific HRQOL questionnaires measure self-perceived health status and functional capacity, while priority instruments include additional aspects such as to have someone to look after the patient or to be a care-giver. This could justify the moderate correlations found, since HRQOL is only part of the priority construct and not its whole constituent. On the other hand, generic HRQOL instruments show little influence by specific diseases such as cataracts. The unexpected weak correlation between the overall priority score of the AI and the anxiety/depression EQ-5D dimension, could be due to the association between mood disturbance and pain and disability among people living with hip and knee osteoarthritis. However, this weak correlation was not observed between the overall priority score of the AI and the dimension on emotion of the HUI3, which also gathers information on anxiety and depression.

In New Zealand and Canada the results from the validation of their prioritisation instruments were similar to those of this study, even though the method followed in their development was different from that of CAHTA's instruments, which reinforces their validity [4,10]. In New Zealand the AI presented weak correlations both with the EQ-5D (0.23) and with the WOMAC (0.26) [24,25,32]. For the CI, the correlations between the priority score and the opinion of the specialist measured through a VAS (0.41) [33] and the VF-14 (0.45) were moderate, and low (0.22) for the EQ-5D [24,25]. In Canada the correlation found between the AI and the opinion of the specialist measured by VAS was strong (0.79), and moderate with the EQ-5D (0.33) and the WOMAC (0.33). In the case of the CI, the correlation with the opinion of the specialist was strong (0.65), weak with the EQ-5D (0.16), and moderate (0.35) with a modified version of the VF-14 [26,27].

The degree of agreement between doctors when evaluating different hypothetical scenarios with the prioritisation instruments was acceptable. Both instruments presented inter-observer reliability values considered appropriate for group comparisons, but the priority score should be interpreted cautiously at the individual level. Although the variability in assessing the priority of patients might exist, overall health resources would be assigned first to those who need them most. In the case of Canada the results were again similar to those found in our study. The ICC for each of the seven criteria of the prioritisation instruments for hip and knee arthroplasties ranged from 0.56 to 0.81 [12].

Limitations of the study include the use of two different sources of information to validate the prioritisation instruments – the doctor and the patient- and doctors answering the VAS as well as administering the prioritisation instrument. The results could have been influenced by the quality of doctor-patient communication and by the perception of each about the health status and functional limitations. In any case, prioritisation instruments are intended to be used in the clinical setting and the correlations observed between the prioritisation instruments and the HRQOL questionnaires, show that doctors are reflecting patients' perceptions in the prioritisation instruments. The fact that doctors answered the VAS and also administered the prioritisation instrument may have affected the results on the correlations between the VAS and the prioritisation instruments, but they were similar to other studies. On the other hand, the lack of information on the doctor who assessed the priority has made it impossible to analyse the variability of the VAS assessments, as was done in New Zealand [33].

The process of patients' recruitment, doctors' participation and the time elapsed between patients' recruitment and the telephone interview are also limitations of the study. Patients were probably not consecutive. However, the priority distribution was similar to that available at the Catalan Health Service for patients in waiting list. In addition, workload was the main reason why a patient was not included in the study which could be assume as a random selection of patients. Not all the doctors invited to participate finally collaborated in the study. If participating doctors collected information in a more accurate manner than would have done the rest of the doctors, our results could have been biased towards higher correlation coefficients and differences between the levels of the priority criteria. On the contrary, the time elapsed between patients' recruitment and the telephone interview might have reduced correlation coefficients. Doctors' perception on patients' functional limitations and pain could have diverged as time went on.

Regarding the reliability analysis, the use of hypothetic scenarios may represent a limitation as it involves a simplified form of real prioritisation. The scenarios had a limited number of patients' characteristics to keep it manageable for participants, which impedes the analysis of patients' characteristics that might have influenced priority assessment. Besides, the scenarios stated clearly which were the patients' priority profiles avoiding other factors that may influence doctor-patient communication during the medical visit, as the influence that patients' requirements and attitudes might have had over doctors' priority assessment. This could have increased the estimation of the prioritisation instruments ICC.

The development of prioritisation instruments could be a time-consuming task, which might induce the utilisation of more simple and quick-to-administer instruments. The strong correlation noted both for the CAHTA's AI and CI with the doctor's opinion on surgical priority could justify VAS utilisation as an alternative prioritisation instrument. Moreover, in Canada the VAS showed an inter-observer reliability of between 0.70 and 0.82 similar to that of CAHTA's AI and CI [12]. However, using this kind of instruments does not make explicit the criteria used to determine the need for surgery, and the instruments themselves may show significant variability [33]. The main contribution of the prioritisation instruments is a greater transparency in the management of waiting lists which may increase trust in the health care system among the general population. Moreover, the inclusion in the prioritisation instruments not only of medical specialists opinions, but also the preferences of general population, patients and close relatives and allied-health professionals, may increase the acceptability of these type of instruments.

The validity of the AI and CI seems acceptable. Besides their construct validity and reliability, the method followed to develop them assures a good content validity, and they also showed an adequate internal validity being able to discriminate between different patients' priority profiles [16,17]. However, there are other important aspects to take into consideration before their implementation. Routine use in the clinical setting requires the instrument to be manageable. Although simplicity could threaten validity, a prioritisation instrument with few criteria reduces the possibility of missing information on some of them. On the other hand, the inclusion in the prioritisation instrument of many criteria usually assessed during the medical visit may reduce the burden of its administration. Nevertheless, the utilisation of these prioritisation instruments could be sometimes difficult. All health professionals and managers must be involved in their implementation. It could be also necessary to supervise its application to prevent distortions in the priority assessment – an inflation of the priority score could happen once patients know which are the criteria used to order the waiting list or doctors could increase the priority of their patients to justify their inclusion in the waiting list-, and it has to be set up as an information system able to both incorporate and apply this new information. However, it is important to highlight that both hip and knee arthroplasties and cataract surgery are surgical interventions that have proved their worth in our society, so that reducing the waiting time for those who most need the surgery implies a significant reduction in the overall burden of impairment and disease [34]. Such a reduction of the burden among the general population could assist families and reduce the costs both of the medical attention required and of the different kinds of social benefits.

## Conclusion

The findings show acceptable validity and reliability for prioritisation instruments in determining the overall priority of a patient awaiting surgery with a high degree of agreement between evaluators. Using prioritisation instruments can bring important improvements in the management of waiting lists.

## Competing interests

The author(s) declare that they have no competing interests.

## Authors' contributions

AA performed the statistical analysis and drafted the manuscript. ME contributed to the design of the study and also drafted the manuscript. MM conducted the field work of the study and collaborated on the draft of the manuscript. MC gave support in the statistical analysis and collaborated on the draft of the manuscript. JMVP collaborated on the draft of the manuscript. All the authors read and approved the final version of the manuscript.

## Pre-publication history

The pre-publication history for this paper can be accessed here:



## Supplementary Material

Additional file 1 Sample scenarios. This file includes two sample scenarios used for the reliability study. Click here for file

## References

[B1] Kelly KD, Voaklander DC, Johnston WC, Suarez-Almazor ME (2002). Equity in waiting times for major joint arthroplasty. Can J Surg.

[B2] Fitzpatrick R, Norquist JM, Reeves BC, Morris RW, Murray DW, Gregg PJ (2004). Equity and need when waiting for total hip replacement surgery. J Eval Clin Pract.

[B3] Arnesen KE, Erikssen J, Stavem K (2002). Gender and socioeconomic status as determinants of waiting time for inpatient surgery in a system with implicit queue management. Health Policy.

[B4] Noseworthy TW, McGurran JJ, Hadorn DC (2003). Waiting for scheduled services in Canada: development of priority-setting scoring systems. J Eval Clin Pract.

[B5] McGurran JJ, Noseworthy TW, and The Steering Committee of the Western Canada Waiting List Project (2002). Improving the management of waiting lists for elective healthcare services. Hosp Q.

[B6] Derrett S, Paul C, Morris JM (1999). Waiting for elective surgery: effects on health-related quality of life. Int J Qual Health Care.

[B7] Mahon JL, Bourne RB, Rorabeck CH, Feeny DH, Stitt L, Webster-Bogaert S (2002). Health-related quality of life and mobility of patients awaiting elective total hip arthroplasty: a prospective study. CMAJ.

[B8] British-Medical-Association, Health Policy and Economic Research Unit of the British Medical Association (1998). Waiting List Prioritisation Scoring Systems: a discussion paper.

[B9] MacCormick AD, Collecutt WG, Parry BR (2003). Prioritizing patients for elective surgery: a systematic review. ANZ J Surg.

[B10] Hadorn DC, Holmes AC (1997). The New Zealand priority criteria project. Part 1: Overview. BMJ.

[B11] Lack A, Edwards RT, Boland A (2000). Weights for waits: lessons from Salisbury. J Health Serv Res Policy.

[B12] Arnett G, Hadorn DC (2003). Developing priority criteria for hip and knee replacement: results from the Western Canada Waiting List Project. Can J Surg.

[B13] Harry LE, Nolan JF, Elender F, Lewis JC (2000). Who gets priority? Waiting list assessment using a scoring system. Ann R Coll Surg Engl.

[B14] Quintana JM, Escobar A, Bilbao A (2006). Explicit criteria for prioritization of cataract surgery. BMC Health Serv Res.

[B15] Escobar A, Quintana JM, Bilbao A, Ibanez B, Arenaza JC, Gutierrez L, Azkarate J, Guenaga JI, Vidaurreta I (2007). Development of explicit criteria for prioritization of hip and knee replacement. J Eval Clin Pract.

[B16] Sampietro-Colom L, Espallargues M, Comas M, Rodriguez E, Castells X, Pinto JL (2006). Prioritizing patients on waiting list for cataract surgery: preference differences among citizens. Gac Sanit.

[B17] Sampietro-Colom L, Espallargues M, Rodriguez E, Comas M, Alonso J, Castells X, Pinto JL (2007). Wide social participation in prioritizing patients on waiting list for joint replacement: a conjoint analysis. Med Decis Making.

[B18] Ryan M, Farrar S (2000). Using conjoint analysis to elicit preferences for health care. BMJ.

[B19] Oudhoff JP, Timmermans DR, Knol DL, Bijnen AB, van der WG (2007). Prioritising patients on surgical waiting lists: a conjoint analysis study on the priority judgements of patients, surgeons, occupational physicians, and general practitioners. Soc Sci Med.

[B20] Ruiz M, Rejas J, Soto J, Pardo A, Rebollo I (2003). Adaptation and validation of the Health Utilities Index Mark 3 into Spanish and correction norms for Spanish population. Med Clin (Barc ).

[B21] Badia X, Roset M, Montserrat S, Herdman M, Segura A (1999). The Spanish version of Euroqol: description and applications. European Quality of Life Scale. Med Clin (Barc).

[B22] Batlle-Gualda E, Esteve-Vives J, Piera MC, Hargreaves R, Cutts J (1999). Translation and adaptation into Spanish of the WOMAC questionnaire specific for hip and knee ostheoarthritis. Rev Esp Reumatol.

[B23] Alonso J, Espallargues M, Andersen TF, Cassard SD, Dunn E, Bernth-Petersen P, Norregaard JC, Black C, Steinberg EP, Anderson GF (1997). International applicability of the VF-14. An index of visual function in patients with cataracts. Ophthalmology.

[B24] Derrett S, Paul C, Herbison P, Williams H (2002). Evaluation of explicit prioritisation for elective surgery: a prospective study. J Health Serv Res Policy.

[B25] Derrett S, Devlin N, Hansen P, Herbison P (2003). Prioritizing patients for elective surgery: a prospective study of clinical priority assessment criteria in New Zealand. Int J Technol Assess Health Care.

[B26] Conner-Spady B, Estey A, Arnett G, Ness K, McGurran J, Bear R, Noseworthy T (2004). Prioritization of patients on waiting lists for hip and knee replacement: validation of a priority criteria tool. Int J Technol Assess Health Care.

[B27] Conner-Spady BL, Sanmugasunderam S, Courtright P, Mildon D, McGurran JJ, Noseworthy TW (2005). The prioritization of patients on waiting lists for cataract surgery: validation of the Western Canada waiting list project cataract priority criteria tool. Ophthalmic Epidemiol.

[B28] Streiner DL, Norman GR, Oxford-University-Press  (2003). Health Measurement Scales A Practical Guide to Their Developement and Use.

[B29] Nunnally JC, Bernstein IR, MacGraw-Hill  (1994). Psychometric theory, 3rd edition.

[B30] Cohen J, Erlbaum L (1988). Statistical power analysis for the behavioral sciences.

[B31] Scientific Advisory Committee of the Medical Outcomes Trust (2002). Assessing health status and quality-of-life instruments: attributes and review criteria. Qual Life Res.

[B32] Coleman B, McChesney S, Twaddle B (2005). Does the priority scoring system for joint replacement really identify those in most need?. N Z Med J.

[B33] Wong VW, Lai TY, Lam PT, Lam DS (2005). Prioritization of cataract surgery: visual analogue scale versus scoring system. ANZ J Surg.

[B34] Williams JI, Llewellyn TH, Arshinoff R, Young N, Naylor CD (1997). The burden of waiting for hip and knee replacements in Ontario. Ontario Hip and Knee Replacement Project Team. J Eval Clin Pract.

