# Supplementary material for: Prioritisation of patients on waiting lists for hip and knee arthroplasties and cataract surgery: Instruments validation
Source: BMC Health Serv Res. 2008 Apr 8;8:76. doi: 10.1186/1472-6963-8-76 (PMC2373288; doi:10.1186/1472-6963-8-76)
Supplement: Additional file 1 — Sample scenarios. This file includes two sample scenarios used for the reliability study. [file 1472-6963-8-76-S1.doc]

# Sample scenarios

## Cataract instrument sample scenario

Male patient 71 years old.

Pathological antecedents without interest.

Ocular examination

Right eye: nuclear cataract, cortical. Corrected visual acuity: 0.20.

Left eye: nuclear cataract, cortical. Corrected visual acuity: 0.40.

There are not ocular comorbidities.

Activities of daily living. He is unable to read small letters, he has a lot of difficulty to read the paper or to do crosswords, but he has not a lot of difficulty to see the signs in the street or the shop windows when he goes for a walk during the afternoon. He neither has a lot of difficulty to watch TV nor to recognise people when they are near him.

Work situation. He retired 8 years ago, but not due to health reasons. He lives with his wife, to whom he helps sporadically in the domestic duties and to do the shopping for the house. He does not has anyone to take care of.

## Hip and knee arthroplasty sample scenario

Male patient 73 years old, who has had discomfort in his left hip for more than 15 years. He has not needed analgesic medication yet. He currently presents moderate pain when he starts walking, range of walking below 500 metres, important limp, he needs the handrail to walk stairs up and down and he has a lot of difficulty to put his shoes on and to stand up. He needs a walking stick for ambulation.

Antecedents: prostatic pathology.

Physical exploration of the left hip:

| Flexion | Extension | Abduction | Adduction | External rotation | Interna rotation |
| --- | --- | --- | --- | --- | --- |
| 80º | 0º | 30º | 10º | 10º | -10º |

Radiology: left hip osteoarthritis, grade IV of Lawrence – Kellgren classification.

Work situation. He retired 8 years ago. He lives with his wife, to whom he occasionally helps in the domestic duties and to do the shopping for the house. He does not has anyone to take care of.
